# Does Steric Hindrance Actually Govern the Competition between Bimolecular Substitution and Elimination Reactions?

**DOI:** 10.1021/acs.jpca.2c00415

**Published:** 2022-03-15

**Authors:** Miguel Gallegos, Aurora Costales, Ángel Martín Pendás

**Affiliations:** Department of Analytical and Physical Chemistry, University of Oviedo, E-33006 Oviedo, Spain

## Abstract

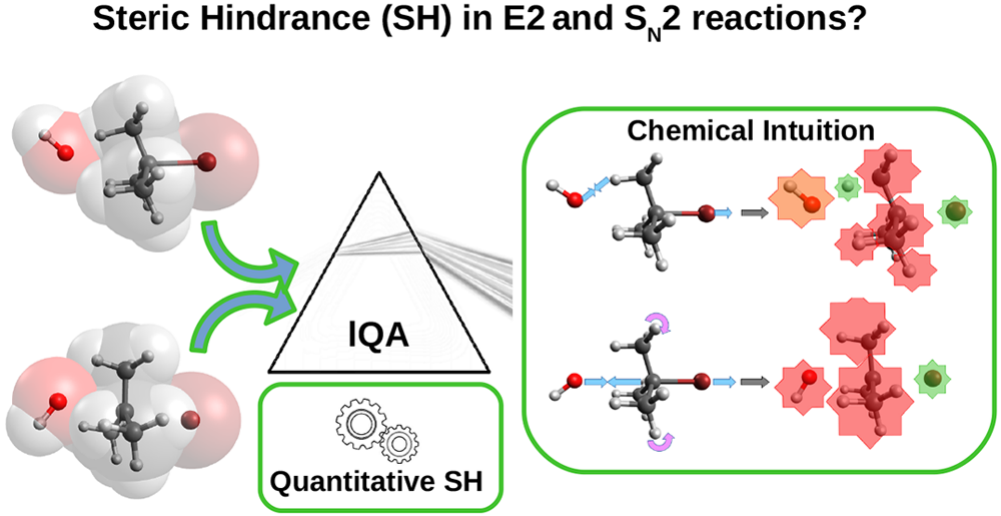

Bimolecular nucleophilic substitution (S_N_2) and elimination (E2) reactions are prototypical examples of competing reaction mechanisms, with fundamental implications in modern chemical synthesis. Steric hindrance (SH) is often considered to be one of the dominant factors determining the most favorable reaction out of the S_N_2 and E2 pathways. However, the picture provided by classical chemical intuition is inevitably grounded on poorly defined bases. In this work, we try to shed light on the aforementioned problem through the analysis and comparison of the evolution of the steric energy (*E*_ST_), settled within the IQA scheme and experienced along both reaction mechanisms. For such a purpose, the substitution and elimination reactions of a collection of alkyl bromides (R-Br) with the hydroxide anion (OH^–^) were studied in the gas phase at the M06-2X/aug-cc-pVDZ level of theory. The results show that, generally, *E*_ST_ recovers the appealing trends already anticipated by chemical intuition and organic chemistry, supporting the role that SH is classically claimed to play in the competition between S_N_2 and E2 reactions.

## Introduction

Substitution (S) and elimination (E) reactions of common carbon-based alkanes (C–X) are, without a doubt, two of the most fundamental chemical processes making up the basic core of classical organic chemistry, being archetypal textbook examples of chemical reactivity.^1,2^ The former process involves, generally, the direct attack of an electrophilic scaffold (El) by a nucleophile (Nu), yielding the corresponding substitution product. On the other hand, the latter comprises the abstraction of an acidic species, such as a H atom vicinal to an electronegative moiety (X), by the nucleophile acting now as a base (B), resulting in the formation of a carbon–carbon double bond (C=C) that is characteristic of the final alkene product. Both chemical transformations lead to the extrusion of a secondary species, the leaving group (L). Despite their simplicity, nucleophilic substitution and elimination reactions are extremely useful in chemistry, having tremendous applications in modern synthetic chemistry and biochemistry^3−6^ such as the well-known Williamson ether synthesis^2,7^ or the Peterson olefination,^8^ to name just a few. Although several mechanisms have been classically proposed for these two reactions,^2^ one of the most common ones is the bimolecular nucleophilic substitution or elimination reaction, referred to as S_N_2 or E2, respectively.

**Figure 1:**
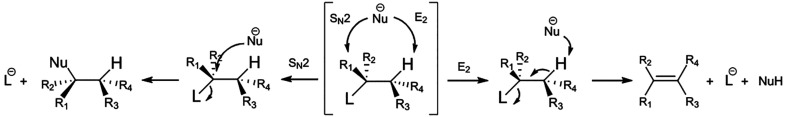
Reaction scheme for the bimolecular substitution and elimination reactions under study.

The typical electron push–pull scheme used to understand these two reaction mechanisms is shown in Figure 1. It should be noticed that in the particular case of the E2 mechanism shown here, the leaving group and H atom at the β-C are considered to be arranged in the usual anticoplanar disposition, although the less common and usually less energetically favorable syn elimination can also be observed in certain scenarios. As can be seen, both bimolecular transformations are assumed to proceed in a concerted or quasi-concerted way, exhibiting almost simultaneous bond formation and cleavage processes and thus being stereospecific. Despite their relative simplicity, the huge conceptual implications and applications of these chemical transformations have led to their extensive study both computationally^9−15^ and experimentally.^16−21^ It is generally accepted that, under common conditions, these two reaction mechanisms are always in competition, as reflected by the ratio of the alkene and substituted-alkane products observed, usually in the final reaction outcome. Therefore and considering the already-mentioned role that S_N_2 and E2 reactions play in modern chemistry, the factors determining their competition have been widely studied in the literature in multiple scenarios.^22−32^ The huge amount of available literature regarding this topic makes it impossible to outline all of the relevant work in the present article. However, it is particularly important to highlight the contributions of Bachrach^33^ and Gronert,^34,35^ among others. Within this context, the driving forces governing the most favorable pathway between these two are generally considered^2^ to arise as a result of the interplay between different factors, including the solvent, the temperature, and the Lewis basicity of the nucleophile/base as recently discussed by Méndez and co-workers,^36^ along with steric effects, among others. For example, polar protic solvents are known to generally favor elimination over substitution reactions as a result of the decrease observed in the nucleophilicity of the attacking species. Such a result is classically claimed to arise from the increase in the relative “bulkiness” of the Nu, embedded in a sphere of H-bonded solvent molecules, which makes it difficult for the latter to approach the C atom of the electrophile, thus favoring the alternative proton abstraction inherent to the E2 reaction. Indeed, the latter concept, steric hindrance (SH), has been used to derive very appealing arguments that are apparently able to explain the origin behind multiple chemical phenomena, including the chemopreference between both reaction mechanisms.^2^ For instance, Brown and co-workers have extensively studied^37−39^ the impact of steric repulsion in elimination reactions, particularly its role in the ratio of the Saytzev and Hofmann alkene products, suggesting that the “Hofmann rule” arises as a direct manifestation of steric effects experienced in the TS structure along elimination reactions, something which has also been studied in cyclic systems,^40^ where strain effects are claimed to be crucial for the anti- or syn- Hofmann eliminations. Moreover and from a classical perspective, it is generally considered that bulkier bases and electrophilic skeletons are more likely to undergo elimination reactions (E) than their less-hindered analogs, whereas the combination of “naked” nucleophiles and poorly substituted scaffolds is more prone to exhibit substitution reactions (S). Such a rationale is directly built on steric arguments: in elimination reactions, the nucleophile or base experiences only very subtle clashing arising from the local, and nearly invariant with the nature of the electrophile, chemical environment faced by it throughout the proton abstraction process. On the other hand, the nucleophilic attack on a much more sterically shielded carbon atom implies that, during substitution reactions, the nucleophile suffers from considerable steric penalties resulting from the congestion against the bulky substituents directly bonded to the electrophilic center. Despite being very appealing, the diffuse nature inherent to those terms crystallizing out of chemical intuition, such as SH, inevitably implies that there is no guarantee that the previous arguments are indeed robust and faithful, so they may actually be built on quicksand. This is not unique to this particular scenario, but rather a lot of debate has appeared in the literature in recent years about whether steric congestion lies behind some commonly observed chemical phenomena, such as rotational barriers^41−43^ and the intriguing conformational stability of some species.^44−49^ Thus, the aforementioned, and similar, problems and controversies have motivated the development and application of the most rigorous tools and methodologies within the chemical sciences. Indeed, this has crystallized in the very prominent implementation of theoretical and computational chemistry in state-of-the-art research of chemical reactivity. As far as SH is regarded, many different attempts have recently been reported to study its influence and role in chemistry^50−55^ under the magnifying glass of different theoretical frameworks. Some examples may include the so-called natural steric analysis, built on the natural bond orbital (NBO) method,^56^ symmetry-adapted perturbation theory (SAPT),^57^ quantum mechanical size as developed by Hollett and co-workers,^58^ and energetic partitioning schemes such as the interacting quantum atoms (IQA)^59^ and the energetic decomposition analysis (EDA)^60^ approaches. Though some of these strategies have been successfully utilized to explain the role of SH in a wide range of chemical phenomena, the picture provided by some of them, especially within the EDA and other path-dependent schemes, may be prone to inconsistencies and incorrect definitions, which still limits the chemical understanding which can be distilled from their application. Fortunately, in one of our previous contributions^61^ we showed that, as already anticipated by Popelier and co-workers,^55^ the IQA energetic partitioning scheme offers an accurate estimator of steric hindrance, the steric energy *E*_ST_, which is able to provide a picture in good agreement with classical chemical intuition that is valid for any general scenario.

Following the previous trends and considering the relevance that SH is claimed to play in common chemical transformations, in this work we try to elucidate whether there is a large difference in the steric clashing experienced along common bimolecular elimination (E2) and substitution (S_N_2) reactions over carbon-based electrophiles, paying special attention to the local steric penalty observed by both the nucleophile/base and the organic scaffold. For such a purpose, the gas-phase reaction between a collection of simple bromo-alkanes (R–Br) and the hydroxide anion (OH^–^) was selected as a test bed model. It should be noticed that although in most applications the S_N_2 and E2 reactions are performed in solution, it has been suggested^35^ that the interplay of the different factors governing the competition among substitution and elimination reactions should be equivalent in solution and in the gas phase, and hence the latter comprises a suitable test bed model system for our study. Indeed, gas-phase calculations have been commonly employed in the recent literature to study these and similar systems.^12,23,30,33,62−65^ Moreover, the actual role of solvation in the competition of the S_N_2 and E2 mechanisms is still a matter of debate,^66^ being a far from trivial topic. Altogether, the impact of solvent effects in the competition between both mechanisms is out of the scope of this work, so gas-phase calculations will be used. This work is organized as follows: First, the real-space steric energy (*E*_ST_) term is introduced. Then, a general overview of the substitution and elimination reaction mechanism is presented. And finally, the evolution of the SH undergone by the different fragments involved in both reaction mechanisms is shown and discussed. The final section gathers the conclusions derived from this work.

## IQA Steric Energy as a Measure of SH

Within the chemistry realm, SH is often considered to arise as a repulsive contribution to the interaction between two chemical systems, supposedly being a direct manifestation of the volume that atoms occupy in physical space. However, as appealing and useful as the classical definition of SH may be, its lack of rigor has motivated its redefinition from numerous theoretical perspectives.^9,55,58,67,68^ Unfortunately, most of the aforementioned attempts have been constructed almost entirely in orbital space, whereas an appealing yet solid estimation of SH inevitably requires a real-space description.

The interacting quantum atoms (IQA) energetic partitioning scheme,^59^ derived within the quantum theory of atoms in molecules (QTAIM)^69^ theory, is a real-space technique which partitions the total energy of a system as
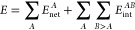
1where the terms *E*_net_^*A*^ and *E*_int_^*AB*^ account for the intra-atomic energy of a QTAIM basin (Ω_*A*_) and the interaction two-body term between a pair of domains (Ω_*A*_ and Ω_*B*_), respectively. Both intra- and interbasin energies can be further written as a sum of physically meaningful terms^59^ whose discussion will be omitted from the current work for the sake of simplicity. Among these two contributions, the change in the net or self-energies (*E*_net_) upon compression, frequently measured with respect to a reference to energy *E*_net_^0^, leads to the so-called deformation energies
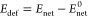
2which have been proven^55^ to provide a suitable estimation of the steric clashing undergone by a chemical system. Despite being valid in many scenarios, deformation energies are heavily dependent on the electron count of a QTAIM domain, which inevitably biases the whole picture provided by them. We have recently proven^61^ that a more truthful estimator of SH, the steric energy *E*_ST_, can be distilled from plain deformation energies by removing the charge-transfer energy contribution, *E*_CT_, as
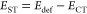
3where the *E*_CT_ term can be readily computed within grand canonical density functional theory in terms of the ionization cost as measured by the ionization potential (IP). Altogether, steric energies have been shown to provide a general and faithful description of steric effects, even in scenarios prone to exhibit significant electronic redistribution.^61^ For such a reason, steric energies will be used as a measure of SH in the current work. It should be emphasized that, owing to the novelty of the *E*_ST_ descriptor, it is not fully addressed whether the latter entirely matches the picture provided by chemical intuition. However, the first results have shown^61^ that it is only the “static” description of steric hindrance, that used to classify a system as being bulky or strained on its own, the one that is not covered by the *E*_ST_ energy. This should not be viewed as a drawback of our approximation but rather ass proof of its consistency: since the definition of *E*_ST_ inevitably requires a reference, the steric clashing or compression of a system is not an absolute but rather a relative quantity, being defined only for an evolving system, such as those found in this work.

## Computational Details

All calculations were performed in the gas phase at the M06-2X/aug-cc-pVDZ level of theory. The equilibrium geometries of the species participating in the reactions were characterized as minima or first-order saddle points through the analysis of the characteristic eigenvalues of the Hessian matrix. Geometry optimizations, frequency calculations, and wave function generations were performed using the *Gaussian 09* quantum chemistry package.^70^ Similarly, IQA calculations were computed using the in-house-developed PROMOLDEN code.^71^ (See SI section 1 for further details.) The reaction energy profiles and the progress of the different energy terms analyzed throughout the reaction are reported many times in terms of the relative ratio or percentage of the reaction coordinate (χ). In all cases, the starting reactant complex, involving the original halo-alkane, was used as a reference for the computations of the deformation (*E*_def_) and steric (*E*_ST_) energies. Moreover, in the case of elimination reactions, the steric hindrance of the H atom being abstracted will be analyzed independently from the remaining organic skeleton. Thus, in the E2 mechanism, the electrophile (El) accounts for all of the atoms of the substrate except for the acidic H atom. Finally and for the sake of convenience, the following color code will be used for the different electrophilic skeletons: red, [CH_3_CH_2_−]; blue, [(CH_3_)_2_CH−]; and green, [(CH_3_)_3_C−]. When comparing trends along progressively bulkier species, unless otherwise specified, the values will be reported in the following order: primary-, secondary-, and tertiary-substituted electrophiles.

## Results and Discussion

### Classical Picture of the S_N_2 and E2 Reactions

**Figure 2:**
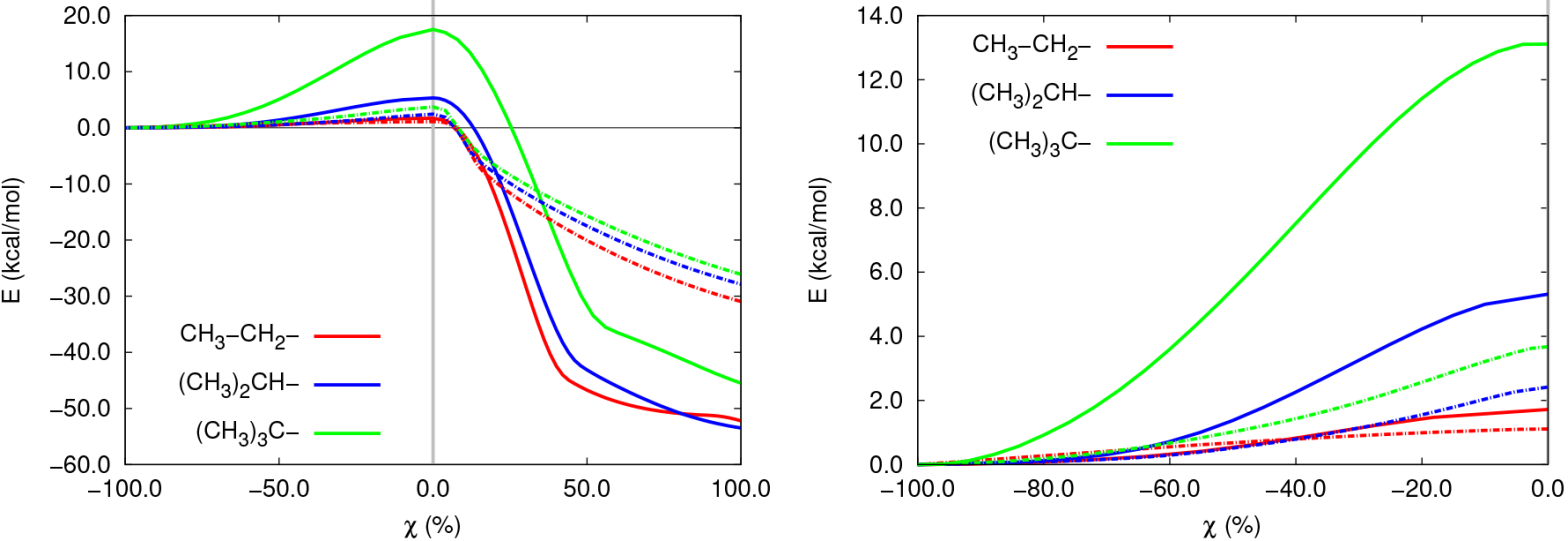
Energy profiles of the gas-phase substitution and elimination reactions under study (left) along with a close-up, showing the transition from the staring reactive complex to the TS (right). Solid and dashed lines are employed to indicate the S_N_2 and E2 processes, respectively.

Before analyzing in detail the evolution of the SH experienced along the substitution and elimination reactions, it is interesting to briefly show and discuss their general features. Figure 2 collects the energy profiles of the gas-phase S_N_2 and E2 reactions under study for different electrophiles.

**Figure 3:**
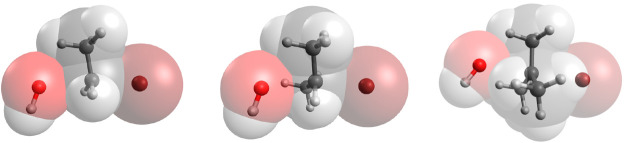
Transition states for the gas-phase substitution reactions with different electrophiles.

**Figure 4:**
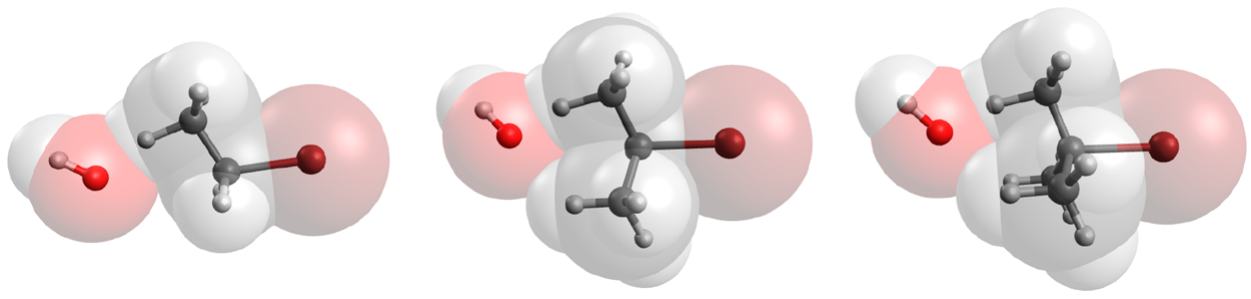
Transition states for the gas-phase elimination reactions with different electrophiles.

As can be seen from Figure 2, all of the studied reactions are exothermic, with Δ*E* values on the order of −50.0 and −30 kcal/mol for S_N_2 and E2, respectively. Such a finding suggests that the TS structures resemble the geometries found in the starting reactive complex, in agreement with the well-known Hammond postulates.^2^ Indeed, the previous observation is clearly reflected in Figures 3 and 4, collecting the optimized geometries of the involved transition states. Furthermore, there is a very clear and interesting trend in the evolution of the reaction energy profiles, particularly as far as the activation energies are regarded. First, as the bulkiness of the central electrophilic carbon atom increases, so does the activation barriers (Δ*E*^act^) of all of the studied processes, a result which holds for both substitution (1.8 < 5.2 < 13.0 kcal/mol) and elimination (1.0 < 2.3 < 3.8 kcal/mol) reactions. Moreover, the aforementioned observation is in good agreement with chemical intuition and can be classically argued by steric means: the steric clashing between the reacting moieties increases with the local substitution of the electrophile, resulting in larger steric penalties and, consequently, larger activation barriers. It is interesting, however, that the actual values of the activation barriers, in terms of electronic energies, are significantly larger for the S_N_2 reaction pathway. Indeed, the difference in the reaction barriers of both mechanisms becomes progressively larger as the electrophile is more sterically hindered, as reflected by the aforementioned Δ*E*^act^ values. It should be noticed that the observed trends for the E2 reactions are in partial disagreement with reported data,^32^ according to which the reaction barriers should slightly decrease with further substitution of the electrophile. However, such a result has been suggested^32^ to be strongly dependent on the basicity or steric requirements of the attacking nucleophile, which may explain our obtained results. Nevertheless, the net behavior that is observed is not unexpected and falls within the accepted chemical rationale; therefore, a very appealing explanation can be built on the basis of the differential congestion experienced throughout the different reaction paths:

- In substitution reactions, the attacking hydroxide OH^–^ anion, acting as a nucleophile, has to overcome the local steric shielding attributed to the substituents of the electrophilic scaffold. Thus, as the electrophilic center becomes increasingly crowded, there is a higher congestion of the central C atom, presumably resulting in an increase in the energy cost required to go from the starting reactive complex geometry to the activated complex structure. Moreover, such a fact can also be used to explain why the ease of substitutions reactions is specially susceptible to the environment of the geminal groups directly attached to the reactive C atom.
- In elimination reactions, the OH^–^ anion acts as a base and consequently experiences only a very subtle steric penalty while leading to proton abstraction, which is characteristic of the elimination process. Similarly, this argument inevitably implies that the local congestion experienced by the OH^–^ anion will be considerably smaller than that undergone through substitution reactions and will be less affected by the local environment of the C atom because the latter is not directly facing the attacking OH^–^. Indeed, it is worth mentioning that all of the elimination reactions studied here show almost equivalent evolutions of both the total reaction energies and reaction forces (SI section 5). Hence, this apparent “invariance” with the bulkiness of the electrophilic skeleton is satisfying within the picture provided by chemical intuition, supposedly arising as a result of the almost identical “local” environment involved in the proton abstraction and planarization processes.

It should be noticed that, at first glance, the observed trends in the activation energies are in good agreement with the picture provided by classical organic chemistry: the SH faced through the S_N_2 reaction increases significantly with the bulkiness of the electrophile, something that occurs via the coupling to the nearly invariant SH through the competitive E2 reaction, and favors the latter for larger organic scaffolds. The previous arguments, which have been questioned in recent years,^32^ are absolutely appealing and are in good agreement with classical organic chemistry. Nevertheless, even though such a rationale can be used to explain the previously observed trends, it is beyond question that it may actually be built on quicksand. Consequently, despite being oddly satisfying, a pure classical steric argument provides only a partial and probably distorted picture of reality, masking the role of other more subtle driving forces. In this work, we will try to shed light on the proposed rationale behind the rules governing the competition between substitution and elimination processes, paying particular attention to the evolution of the steric congestion truly experienced by the different chemical moieties participating in the already-discussed reactions.

### *E*_ST_ throughout the Reactions

**Figure 5:**
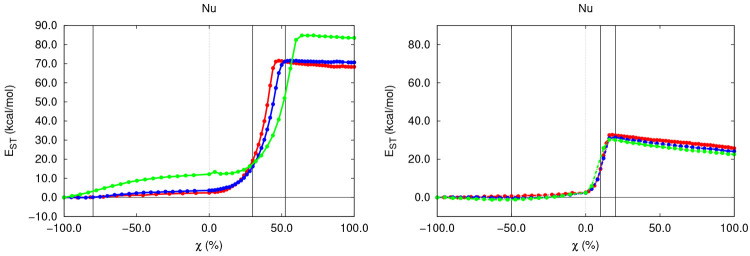
*E*_ST_^OH^ along the S_N_2 (left) and E2 (right) gas-phase reactions with different substrates.

**Figure 6:**
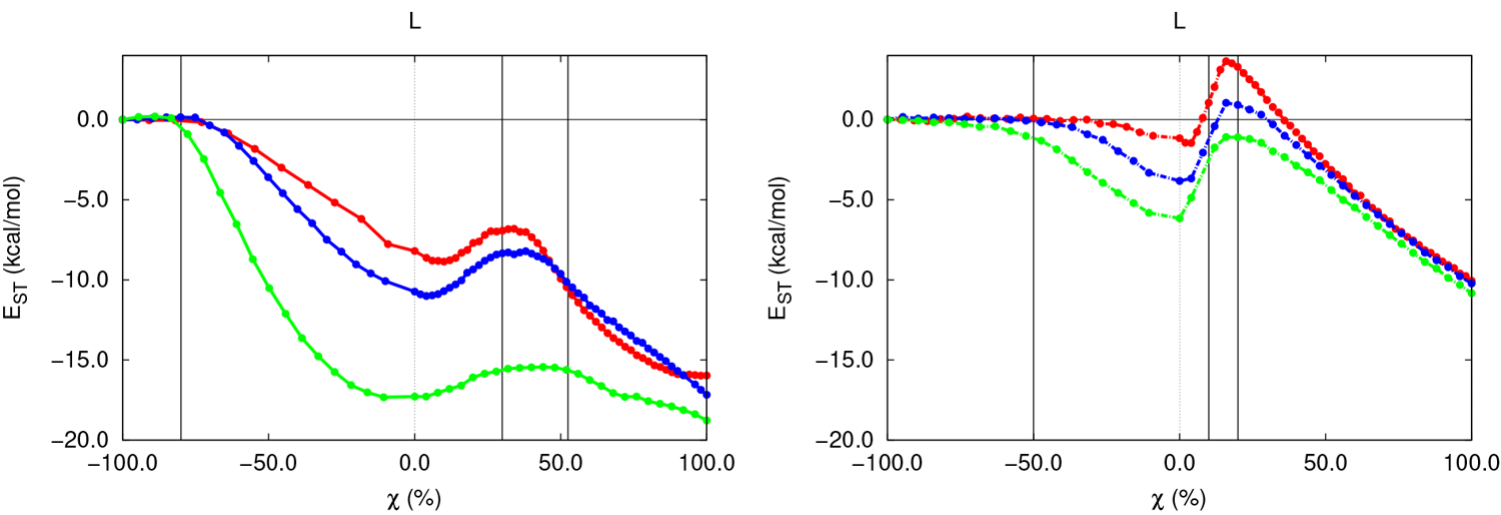
*E*_ST_^Br^ along the S_N_2 (left) and E2 (right) gas-phase reactions with different substrates.

**Figure 7:**
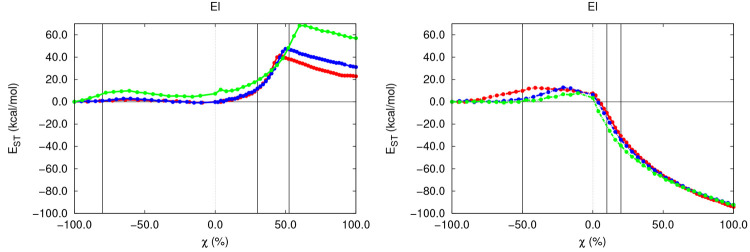
*E*_ST_^El^ along the S_N_2 (left) and E2 (right) gas-phase reactions with different substrates. For E2 reactions, El is reported without the acidic H being abstracted.

Figures 5–7 collect the evolution of the steric energy experienced by the groups involved in the S_N_2 and E2 reactions over different substrates.

**Figure 8:**
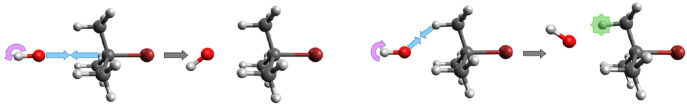
Preparation stage of the S_N_2 (left) and E2 (right) reactions.

**Figure 9:**
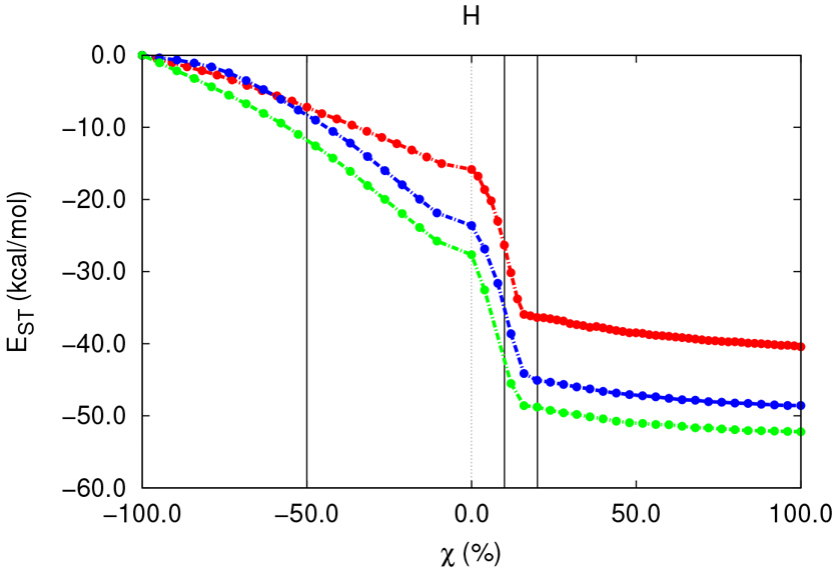
Steric energies of the abstracted H atom along the elimination gas-phase reactions with different substrates.

**Figure 10:**
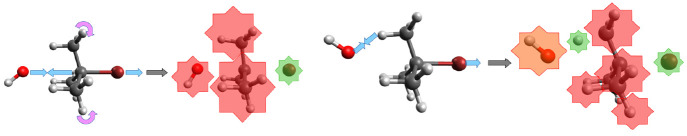
Postpreparation stage of the S_N_2 (left) and E2 (right) reactions.

**Figure 11:**
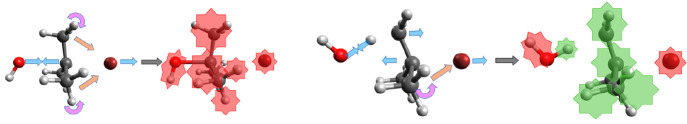
Full bond formation and breaking stage of the S_N_2 (left) and E2 (right) reactions.

**Figure 12:**
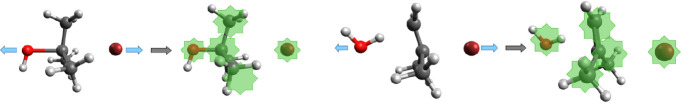
Final reaction stage of the S_N_2 (left) and E2 (right) reactions.

Although both mechanisms are generally considered to be concerted, a careful inspection of the reaction profiles reveals different reaction stages, something which becomes especially pronounced by analyzing the progress of the reaction force experienced. (See SI section 5 for more details.) Thus, it is convenient to discuss the evolution of *E*_ST_ along the different reaction stages involved in the studied chemical transformations, as indicated by the vertical lines shown in Figures 5–7. Moreover and for the sake of clarity, a schematic representation of the major geometrical distortion and the accumulation or relief of SH, as indicated by the red/orange and green regions appearing in Figures 8–12, respectively, will be shown for each of the reaction steps.

- Initially, both reactions involve the OH^–^ anion approaching the core of the organic scaffold (El), as shown in Figure 8.

This preparation step is characterized by an almost negligible evolution of the reaction force (SI section 5) with no significant changes in the local geometries of the El and Nu species, except for the obvious El–Nu distance. This regime, extending up to χ ≈ −80 and −50% for the S_N_2 and E2 reactions, respectively, is characterized by barely no changes in *E*_ST_. Such a result, in agreement with the aforementioned invariance of the geometrical parameters (SI Ssection 2), holds for all of the groups with the particular exception of the acidic H atom along the E2 reaction which, as shown in Figure 9, undergoes a noticeable decongestion, close to −10.0 kcal/mol in the general case, even in this initial reaction stage. Such steric relief in *E*_ST_^H^, which will be generally observed along the entire reaction, comes as a result of the moderate and early elongation of the C–H bond (SI section 2.3). This relieving effect increases with the bulkiness of the starting skeleton, showing limiting values of −7.0, −8.0, and −11.0 kcal/mol for the different substrates. Such a trend arises from the larger “in-molecule” strain to which the H atom is originally exposed in the presence of more crowded organic scaffolds and which will be progressively lost during the elimination reaction as a result of the H-abstraction process.

- Beyond the previous point and up to the TS (χ = 0.00), the ongoing Nu-El compression leads to a much more evident distortion of all the geometrical features of the system, as collected in Figure 10, something which will be accompanied by a significant change in *E*_ST_ for all the groups.

More specifically, this postpreparation stage is characterized by the weakening of the C–Br interaction which will ultimately result in the extrusion of the Br^–^ anion, characteristic of the C–Br bond cleavage and evidenced by the elongation of the latter through both reaction mechanisms (SI section 2.2). Such a lengthening of the C–Br bond naturally leads to a very rapid decrease in *E*_ST_^Br^, as reflected in Figure 6, showing values of −7, −10, and −17 kcal/mol and −1, −4, and −6 kcal/mol, for the substitution and elimination reactions, respectively, taking place over different substrates. This stabilizing trend comes as a result of the decompression undergone by the leaving group, which frees itself from the starting “in-molecule” crowding. Moreover, the decrease in *E*_ST_^Br^ is more pronounced for bulkier substrates, something which accepts an explanation analogous to that provided previously for the steric decompression undergone by the acidic H atom. On the other hand, the decrease in the SH of L is considerably larger for S_N_2 reactions, following the trends observed in the much more prominent elongation of the C–Br bond (0.20, 0.25, and 0.50 Å), when compared with the elimination (E2) pathway (0.05, 0.10, and 0.15 Å). The C–OH and H–OH bond-formation processes taking place simultaneously with this C–Br bond cleavage for S_N_2 and E2 reactions, respectively, start to slowly take place. The further approximation of the nucleophile to the electrophilic center crystallizes in the very slow increase in *E*_ST_^OH^ of less than 5.0 kcal/mol in the general case, as reflected in Figure 5. Such mild and subtle steric congestion is more relevant for substitution reactions, particularly in the case of (CH_3_)_3_CBr, for which the OH^–^ anion experiences a more evident compression (∼10 kcal/mol) arising from the large bulkiness of all of the spectator groups that must be faced by the latter along the back-side attack of the electrophile. On the other hand, the E2 mechanism proceeds with much smaller steric congestion values for the nucleophile along this regime. Indeed, this observation, which will hold for the entire reaction and that is moreover nearly invulnerable to the decoration of the organic scaffold, is in agreement with chemical intuition and arises from the much milder compression faced by the OH^–^ anion, against a “tiny” H atom, during elimination reactions. Additionally, as far as the electrophile is regarded, a very relevant distortion of the organic skeleton takes place along both reaction mechanisms, though drastically different trends can be found along these two mechanisms (Figure 7). In the case of S_N_2 reactions, El undergoes rapid planarization, as reflected by the increase in the internal angle of the latter (SI section 2.4) to yield planar or quasi-planar TS structures characterized by internal angles of about 116, 118, and 121° at χ = 0.00. Interestingly enough, this maximum is not perfectly centered on the TS as a result of the asymmetric compression induced by the different nucleophile and leaving group species. This local distortion of the electrophile, inherent to the characteristic Walden inversion commonly found in S_N_2 reactions, is considerably softer in the case of the E2 reaction, which shows a heavily retarded planarization (111, 114 and 114° at χ = 0.00) (SI section 2.4). This observation, in agreement with chemical intuition, can account for the very minor nucleophile-induced compression of the electrophile accompanying the proton abstraction phenomenon. These findings are, moreover, clearly reflected in the evolution of *E*_ST_^El^, which experiences a moderate increase of less than 20 kcal/mol on going from the reactant complex to the TS structure for both reactions. In either case, the steric stress observed in El is mainly attributed to the conspiring interplay between the penalizing OH^–^ approximation and the relieving Br^–^ extrusion, which overall results in the non-negligible compression of the electrophile. Moreover, the ongoing planarization of El seems to have a forgiving effect on the total steric stress experienced by the latter. This result, being in agreement with already-reported data for the sterically favorable planarization of simple gas-phase alkanes,^72^ would explain the intriguing behavior observed in *E*_ST_^El,S_N_2^ which, despite building up in the very early stages of the reaction, is partially counteracted by the electrophile planarization, leading to small maxima in SH (peaking between 2 and 11 kcal/mol) even before the TS, at roughly χ = −50.0%. However, the much less prominent and late distortion of the El geometry in elimination reactions decreases the favorable effect attributed to the planarization and explains the slow but steady buildup of *E*_ST_^El,E2^ within this regime, arising almost solely from the penalizing OH^–^ compression.

- The previous preparation and distortion stages are followed by the concomitant completion of the bond-formation and bond-breaking processes, leading to the almost fully formed structures of the final products of the reaction, as shown in Figure 11.

Although this stage roughly extends up to χ = 53 and 20% for the S_N_2 and E2 reactions, respectively, slightly different trends can be observed at χ = 30 and 10% for both chemical transformations. The advanced extrusion of the Br^–^ atom accompanying the cleavage of the C–Br bond should intuitively lead to the further decrease in *E*_ST_^Br^; however, this is not the case, as shown in Figure 6, which shows that a moderate compression of L of about 3–5 kcal/mol takes place throughout both reaction mechanisms. This intriguing burst in the SH of the Br^–^ atom is observed along both reaction mechanisms. In the case of the S_N_2 reaction, it arises as a side effect of the Walden inversion which inevitably forces the geminal groups directly attached to the electrophilic C atom toward the leaving group, accompanying the repyramidalization of the organic skeleton, as reflected by the evolution of the internal angle exhibiting values of 108, 114 and 114° (SI section 2.4), and thus leading to a moderate compression of the Br atom. Interestingly enough, a similar effect is observed along elimination reactions, though it arises from a slightly different transformation. In the latter case, the late but abrupt distortion of the local geometry of the electrophile required to achieve the characteristic planarity of a C=C double bond leads to the brief compression of L, induced by the minor rearrangement undergone by the CR_2_ moiety along this transformation. It should be noticed that in both cases this compression rapidly vanishes, being almost immediately counteracted by the further separation of the free Br^–^ from the remaining reaction products, leading once again to the appealing decrease in *E*_ST_^Br^ which will be observed in the remaining stage of the reaction. Additionally, as far as OH^–^ is regarded, the C–OH and H–OH bonds are fully formed during this reaction stage, something which becomes directly evident by the nearly invariant values shown by their respective bond distances beyond this point. (See SI sections 2.1 and 2.3 for more details.) In E2 reactions, the interplay between the full cleavage of the C–H bond and the formation of the OH–H bond leads to a very rapid decompression of the SH suffered by the H atom, *E*_ST_^H^, as reflected by Figure 9. This very prominent decompression of about −20 kcal/mol, measured with respect to the χ = 0.00 point, arises from the full detachment of the H atom from the original organic skeleton at the cost of suffering from a much more subtle compression against the less bulky OH^–^ anion. Analogously, the H–OH compression also leads to a moderate congestion of the latter, as shown in Figure 5, which is almost identical for all of the studied elimination reactions, with a value of Δ*E*_ST_^OH^ ≈ 30 kcal/mol. This observation is in agreement with classical chemical intuition and has a very simple explanation: the local environment seen by the attacking OH^–^ anion throughout the proton abstraction is almost independent of the remaining organic skeleton. On the other hand, in S_N_2 reactions, the formation of the C–OH bond results in a very rapid burst of the SH experienced by the OH^–^ anion (about 70 kcal/mol with respect to the TS), as collected in Figure 5. This result is once again appealing, arising from the steric penalty that has to be faced by the nucleophile to go from a “free” gas-phase species to the “in-molecule” scenario characteristic of the final R–OH substitution product. Moreover, a closer inspection of the evolution of *E*_ST_^OH,S_N_2^ reveals that the OH^–^ anion seems to exhibit larger steric penalties as the electrophile becomes progressively more substituted, something which is especially pronounced for the tertiary substituted species. Furthermore, the SH experienced by the nucleophile is considerably larger than the one experienced throughout the E2 pathway. Indeed, the maxima of the *E*_ST_^OH^ peak are about 70, 70, and 85 kcal/mol and 32, 31, and 30 kcal/mol for the S_N_2 and E2 reactions with different electrophiles, respectively. This observation is in perfect agreement with the chemical rationale and can be explained by taking into account the difference in the steric clash undergone through both reaction mechanisms: whereas in S_N_2 reactions the attacking species experiences a direct steric clash with the electrophilic skeleton, E2 reactions proceed with a more local and subtle clash against a significantly less bulkier H atom. Finally, as far as the electrophile is regarded, drastically different trends are observed in both competing mechanisms. In the case of S_N_2 reactions, the repyramidalization of the central C atom takes place, attributed to the already mentioned Walden inversion. Such an inversion, reflected by the decrease in the internal angle of the electrophile (SI section 2.4), results, along with the compression attributed to the C–OH bond formation, in the steady and smooth increase in *E*_ST_^El,S_N_2^ showing values of 40, 45, and 70 kcal/mol, as collected in Figure 7. These results suggest that the skeleton of the resulting alcohol (R–OH) is more sterically crowded than the starting bromoalkane and that the crowding increases with the substitution of the electrophilic center, in agreement with chemical intuition. On the other hand, *E*_ST_^El,E2^ decreases significantly along the remaining reaction stages, with an Δ*E* value of about −30 to −40 kcal/mol for all of the skeletons. This chemically appealing trend is the result of the later planarization of the electrophile which, together with the Br^–^ and H^+^ extrusions, leads to a very relevant relieving effect of the steric crowding of El. The previous trend will be observed during the remaining reaction stages.

- Finally, the very late stages (up to the reaction completion at χ = 100.0%) of both reaction mechanisms are characterized by a much more subtle evolution of the geometrical parameters, as shown in Figure 12.

This last reaction step is mainly characterized by the spatial separation of the already-formed reaction products, as reflected, for instance, in the trends shown by the C–Br and C–H distances (SI section 2). It is precisely this further long-range separation of the final and fully formed reaction products that leads to a very soft and smooth steric decongestion of all of the species as reflected by the previous figures, in agreement with classical chemical intuition.

## Conclusions

Steric hindrance (SH) has been claimed to be one of the most dominant driving forces governing the competition between S_N_2 and E2 reaction mechanisms. Though appealing and useful, the lack of rigor of chemical intuition weakens the picture and robustness provided by these and other chemical unicorns. In this article, we have used the IQA methodology to study the different steric clashes in the competing nucleophilic substitution and elimination gas-phase reactions between the OH^–^ anion and different alkyl bromides (R–Br). The S_N_2 reactions that are studied are accompanied by considerably larger SH than the E2 analogs, in general agreement with chemical intuition. Additionally, the results obtained suggest that the SH experienced throughout the bimolecular nucleophilic substitution reaction is more sensitive to structural changes in the electrophile when compared to the elimination reaction, a fact in agreement with common organic chemistry textbooks. Altogether and interestingly enough, the results obtained in the present work support most of the classical chemical rationale built around the competition of the S_N_2/E2 mechanisms, showing how steric energies (*E*_ST_) are able to provide highly valuable and quantitative insights into chemical reactivity.

## Supporting Information Available

Relevant results and additional material, such as the equilibrium geometries, reaction forces, atomic charges, different energy terms, and estimated ionization potentials employed for the calculation of the charge-transfer energy contribution (PDF)
