# Supplementary material for: Does Steric Hindrance Actually Govern the Competition between Bimolecular Substitution and Elimination Reactions?
Source: J Phys Chem A. 2022 Mar 15;126(11):1871–80. doi: 10.1021/acs.jpca.2c00415 (PMC8958592; doi:10.1021/acs.jpca.2c00415)
Supplement: Supplementary file 1 — jp2c00415_si_001.pdf [file jp2c00415_si_001.pdf]

Supporting Information

Does Actually Steric Hindrance Govern the Competition between Bi-molecular Substitution and Elimination Reactions?

Miguel Gallegos*, Aurora Costales*, Ángel Martín Pendás*

* Department of Analytical and Physical Chemistry, University of Oviedo, E-33006, Oviedo, Spain.

Contents

1 IQA Computational details **S3**

2 Geometrical Features **S4**
2.1 C(El)-OH distance S4
2.2 C(El)-Br distance S4
2.3 H-OH and C(El)-H distances S5
2.4 Internal Angle of the Electrophile S5

3 Atomic charges **S6**
3.1 Substitution reactions S6
3.2 Elimination reactions S7

4 Deformation energies **S9**

5 Reaction force **S12**

6 Transition State geometries **S13**
6.1 Substitution reactions S13
6.2 Elimination reactions S14

7 Ionization potentials **S16**

8 Color code used along the Supporting Information **S17**

1 IQA Computational details

This section collects the computational details about the IQA energetic partitioning calculations:

All IQA studies collected along the manuscript were performed using the in-house made PROMOLDEN [1] code. The calculations were obtained without accounting for explicit symmetry using β-spheres. A total of 451 points were used in out-β integrations, using Gauss-Legendre radial quadratures with maximum $L = 12$ and 5810 angular Lebedev quadratures. On the other hand, in-β integrations were done using 451 points Gauss-Chebychev radial quadratures and a L value of 10. The Lebedev grid was not changed throughout.

2 Geometrical Features

The current section gathers the evolution of the most relevant geometrical features, measured as relative values with respect to the starting reactive complex used as reference, involved in the reactions under study.

2.1 C(El)-OH distance

The following figures show the evolution of the distance from the OH^- anion to the central C atom of the electrophile (El) throughout the reactions.

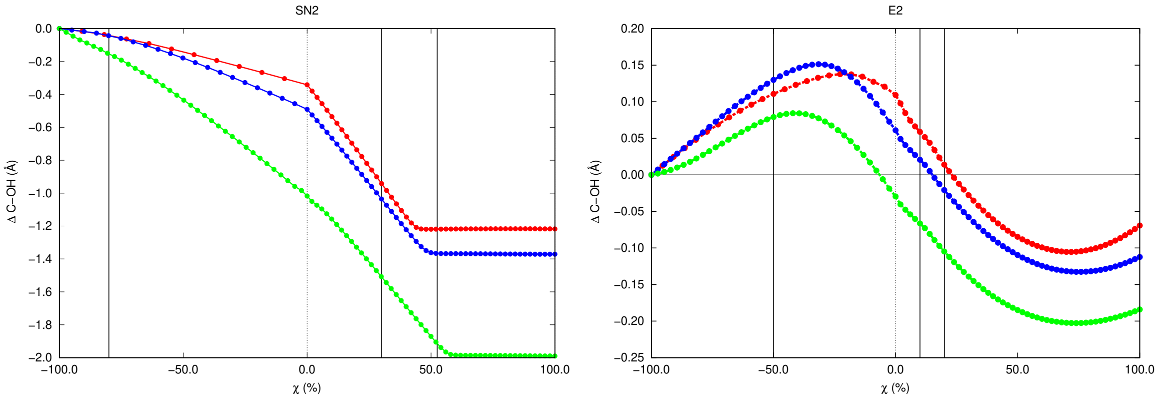

Figure S1: Evolution of the OH-C distance throughout the substitution (left) and elimination (right) reactions. Reference values are: 2.66, 2.81 and 3.43 Å and 3.02, 3.06 and 3.12 Å for the substitution and elimination mechanisms, respectively.

2.2 C(El)-Br distance

The following figures show the evolution of the distance from the Br atom to the central C atom of the electrophile (El) throughout the reactions.

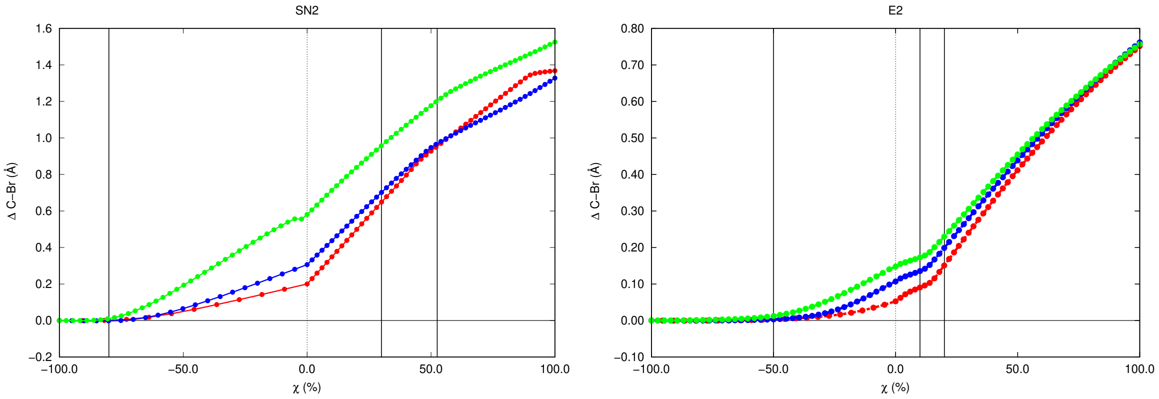

Figure S2: Evolution of the C-Br distance throughout the substitution (left) and elimination (right) reactions. Reference values are: 2.04, 2.05 and 2.04 Å and 2.03, 2.05 and 2.08 Å for the substitution and elimination mechanisms, respectively.

2.3 H-OH and C(El)-H distances

The following figures show the evolution of the distances from the acidic H atom, that is the one being abstracted throughout the elimination reaction, to the attacking OH^- anion (left) and to the central C atom of the electrophile (right).

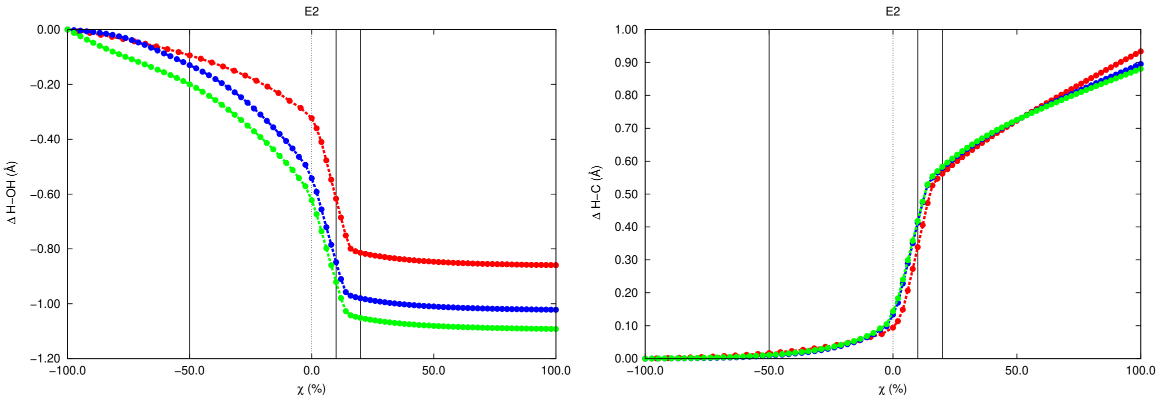

Figure S3: Evolution of the H-OH distance (left) and C-H distance (right) throughout the elimination reactions. Reference values are: 1.84, 2.00 and 2.10 Å and 1.12, 1.11 and 1.11 Å, for the H-OH and C-H distances, respectively.

2.4 Internal Angle of the Electrophile

The following figures show the evolution of the internal angle of the electrophile, measured with respect to the geminal groups directly attached to the central C atom, throughout the reactions. The angel corresponds to that formed by the consecutive geminal atoms to the central atom of the electrophile (C-C-C or C-C-H).

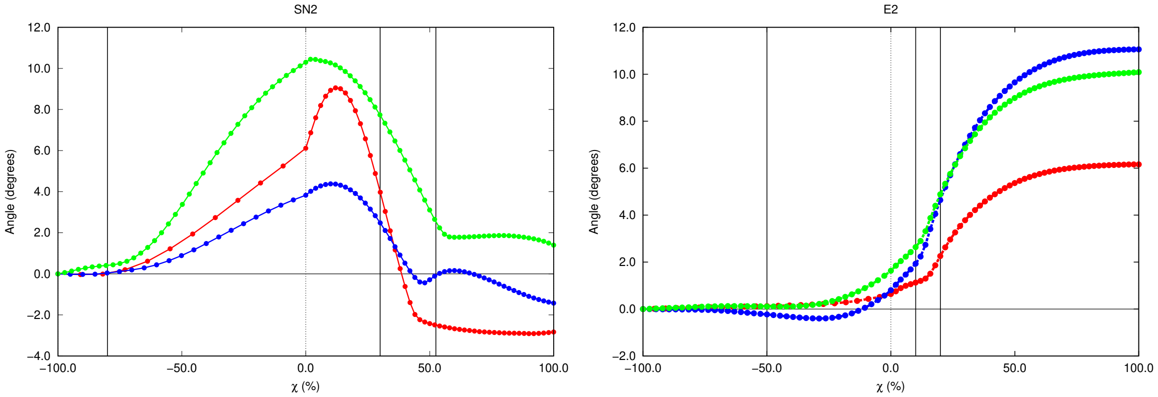

Figure S4: Evolution of the central angle of the electrophilic skeleton throughout the substitution (left) and elimination (right) reactions. Reference values are: 109.8, 113.7 and 111.8 ° and 110.1, 113.7 and 111.9 ° for the substitution and elimination mechanisms, respectively.

3 Atomic charges

The current section gathers the evolution of the atomic charges along the progress of the studied chemical transformations.

3.1 Substitution reactions

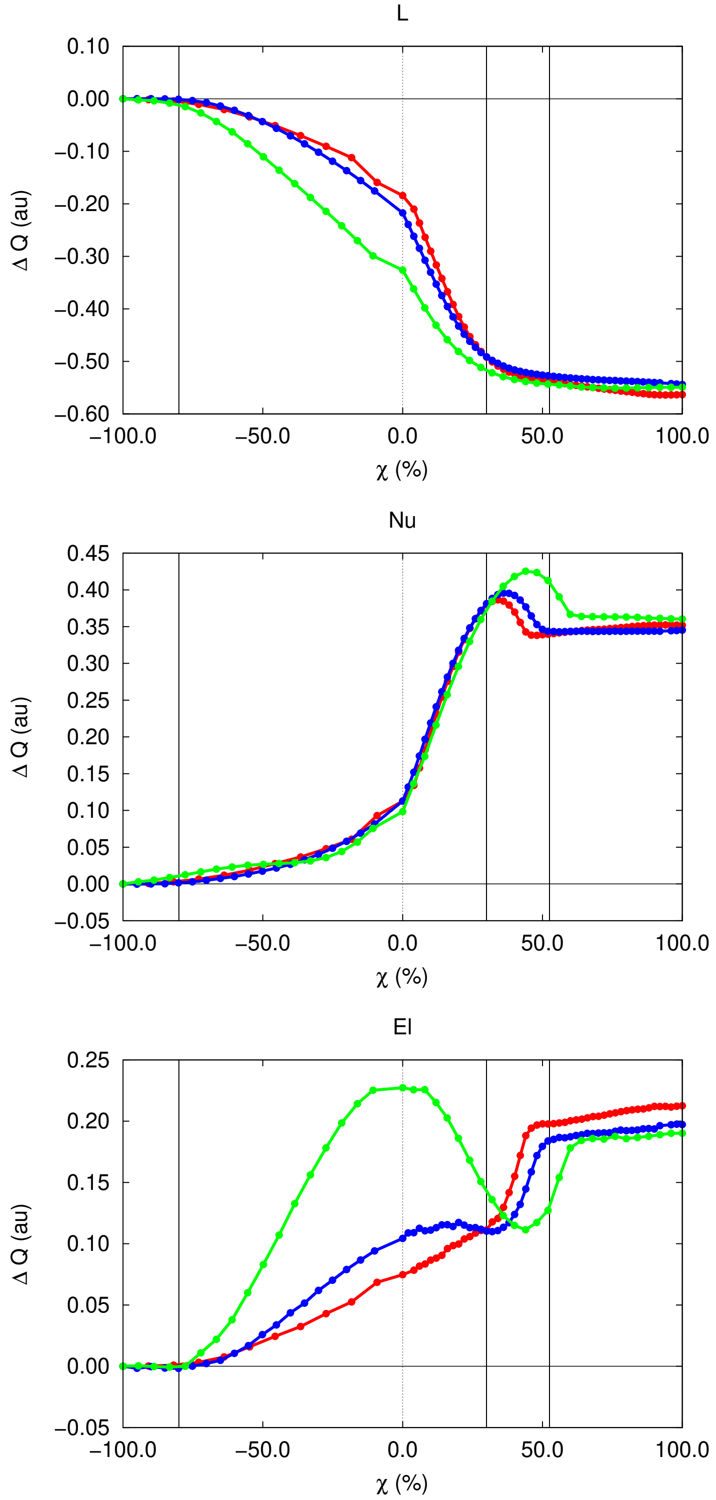

Figure S5: Evolution of the atomic charges of the Leaving group (L), Nucleophile (Nu) and Electrophile (El) throughout the substitution reactions.

3.2 Elimination reactions

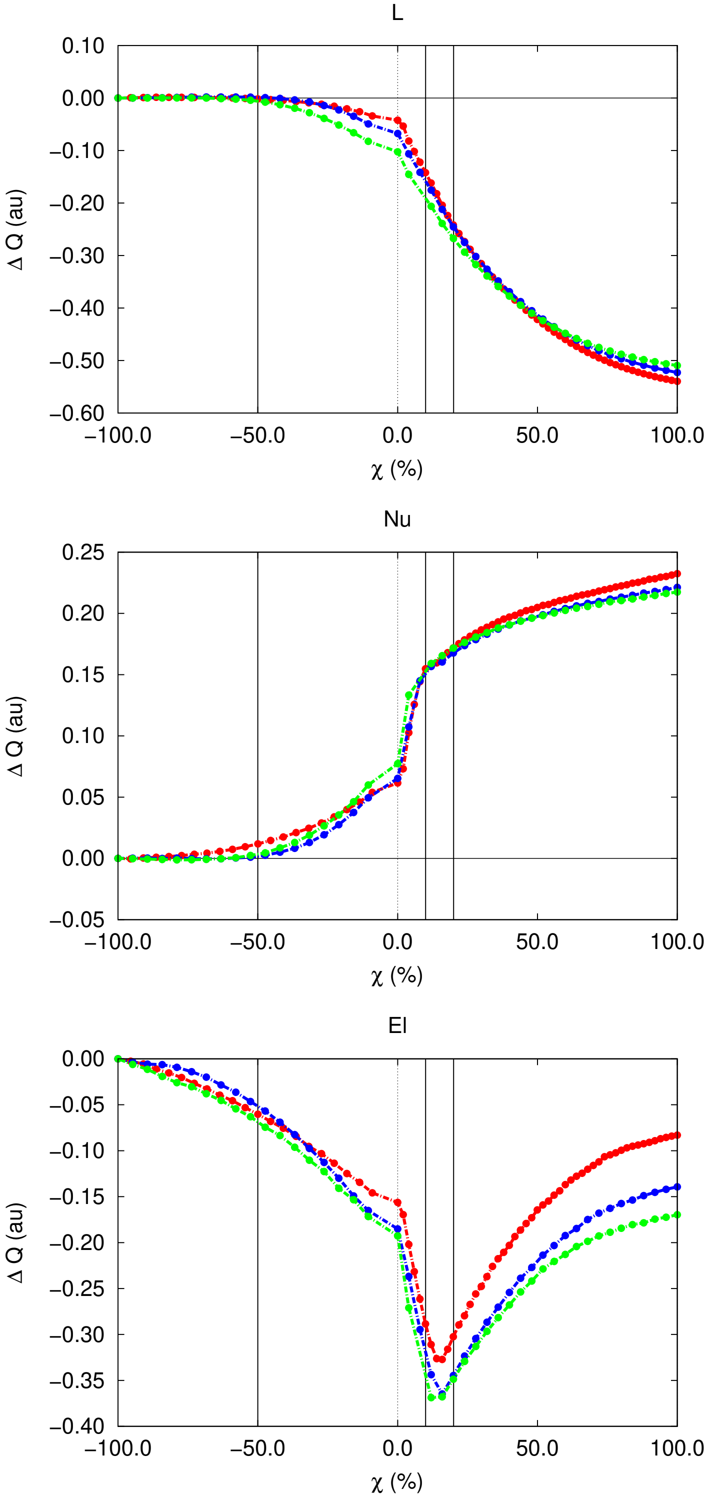

Figure S6: Evolution of the atomic charges of the Leaving group (L), Nucleophile (Nu) and Electrophile (El) throughout the elimination reactions.

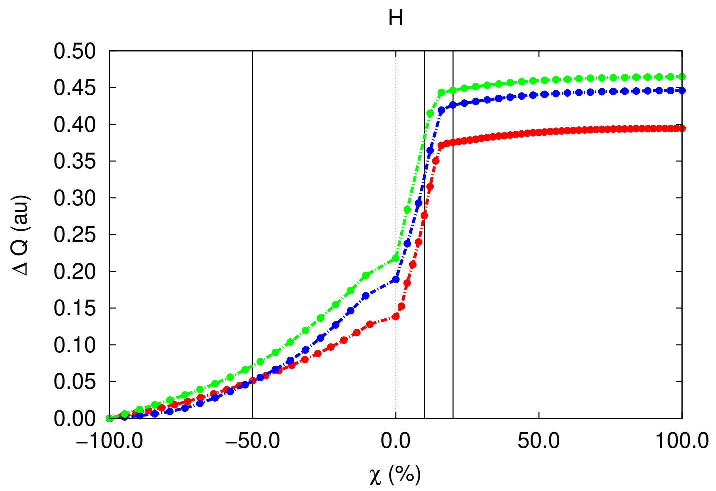

Figure S7: Evolution of the atomic charges of the acidic H atom throughout the elimination reactions.

The following tables collect the reference values of the atomic charges of the relevant groups involved in the substitution and elimination reactions under study.

Substrate	Q(L)	Q(Nu)	Q(El)
CH_3CH_2-	-0.391	-0.925	0.318
$(CH_3)_2CH$-	-0.387	-0.925	0.316
$(CH_3)_3C$-	-0.368	-0.941	0.314

Table S1: Reference value of the atomic charges of the Leaving group (L), Nucleophile (Nu) and Electrophile (El) for the substitution reactions under study. All values are reported in atomic units. Calculations performed at the M06-2X/aug-cc-pVDZ level in the gas phase.

Substrate	Q(L)	Q(Nu)	Q(El)	Q(H)
CH_3CH_2-	-0.365	-0.935	0.074	0.227
$(CH_3)_2CH$-	-0.383	-0.925	0.135	0.173
$(CH_3)_3C$-	-0.399	-0.918	0.168	0.153

Table S2: Reference value of the atomic charges of the Leaving group (L), Nucleophile (Nu), Electrophile (El) and acidic H atom (H) for the elimination reactions under study. All values are reported in atomic units. Calculations performed at the M06-2X/aug-cc-pVDZ level in the gas phase.

4 Deformation energies

This section gathers the evolution of the deformation E_{def} energies along the progress of the chemical reactions under study.

Substitution reactions

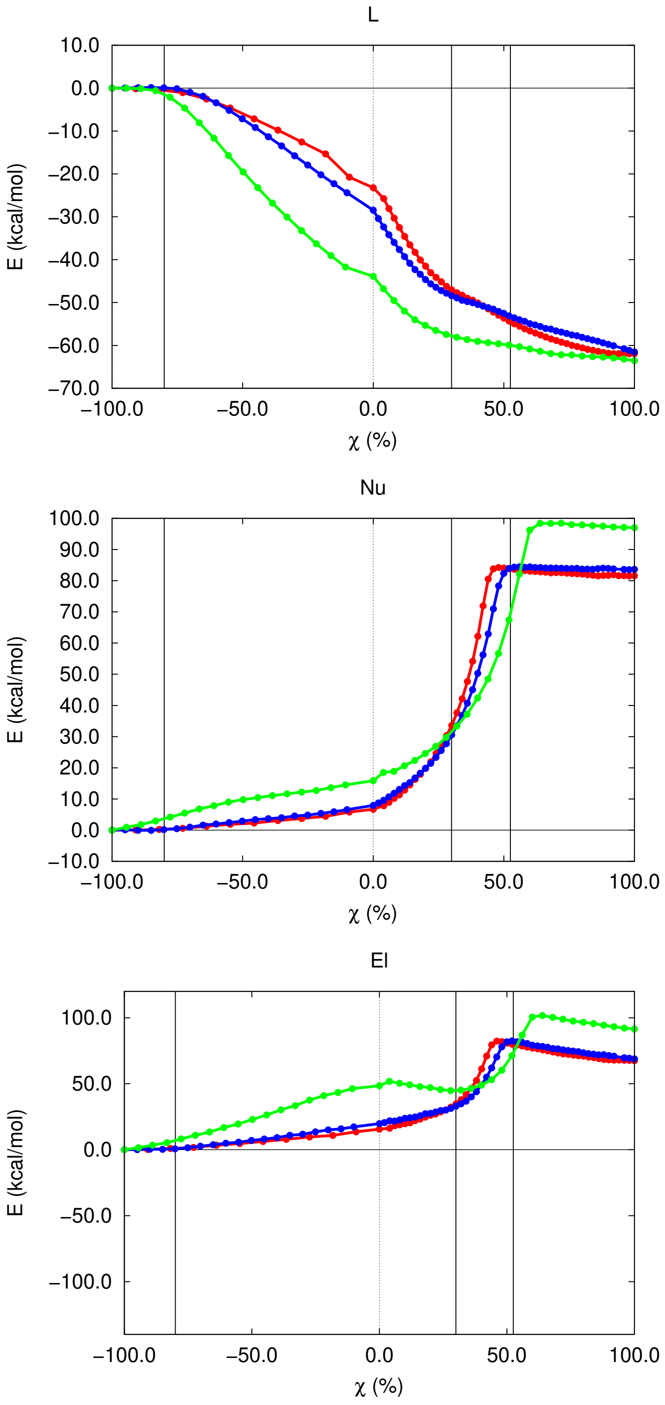

Figure S8: Evolution of the deformation energies of the Leaving group (L), Nucleophile (Nu) and Electrophile (El) throughout the substitution reactions.

Elimination reactions

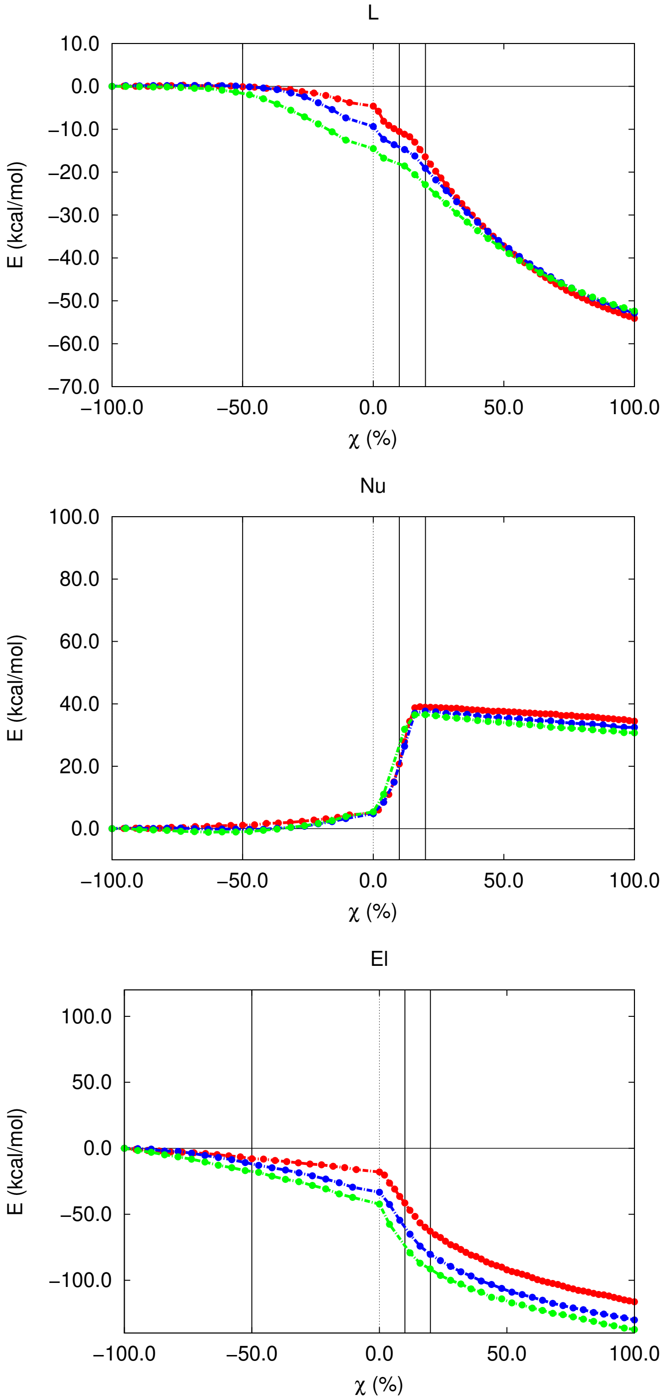

Figure S9: Evolution of the deformation energies of the Leaving group (L), Nucleophile (Nu) and Electrophile (El) throughout the elimination reactions.

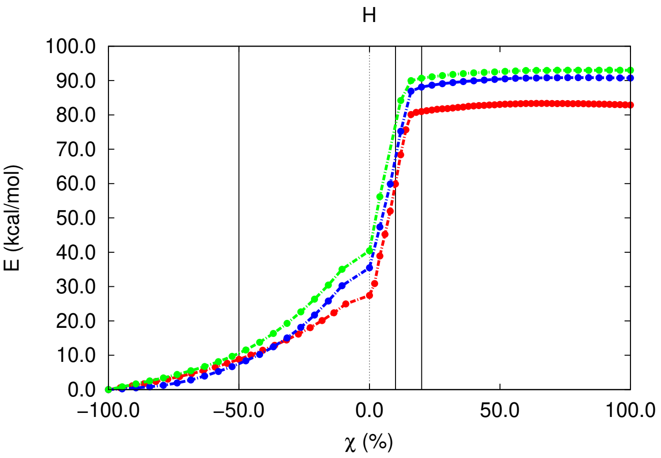

Figure S10: Evolution of the deformation energies of the acidic H atom (H) throughout the elimination reactions.

5 Reaction force

This section collects the evolution of the reaction force throughout the studied chemical transformations with different substrates. The reaction force[2] was originally defined as the negative derivative of the energy with respect to the reaction coordinate (χ), as:

$$F(\chi) = -\frac{dE(\chi)}{d\chi}. \tag{1}$$

For the sake of simplicity, the reaction force values reported along this manuscript were estimated, in an approximate way, through numerical differentiation, as:

$$F(\chi^{ab}) = -\frac{E(\chi^b) - E(\chi^a)}{\chi^b - \chi^a}, \tag{2}$$

where, χ^{ab} is the average value of the reaction coordinate between the points χ^a and χ^b.

The following figures show the evolution of the reaction force along the substitution and elimination reactions over different substrates.

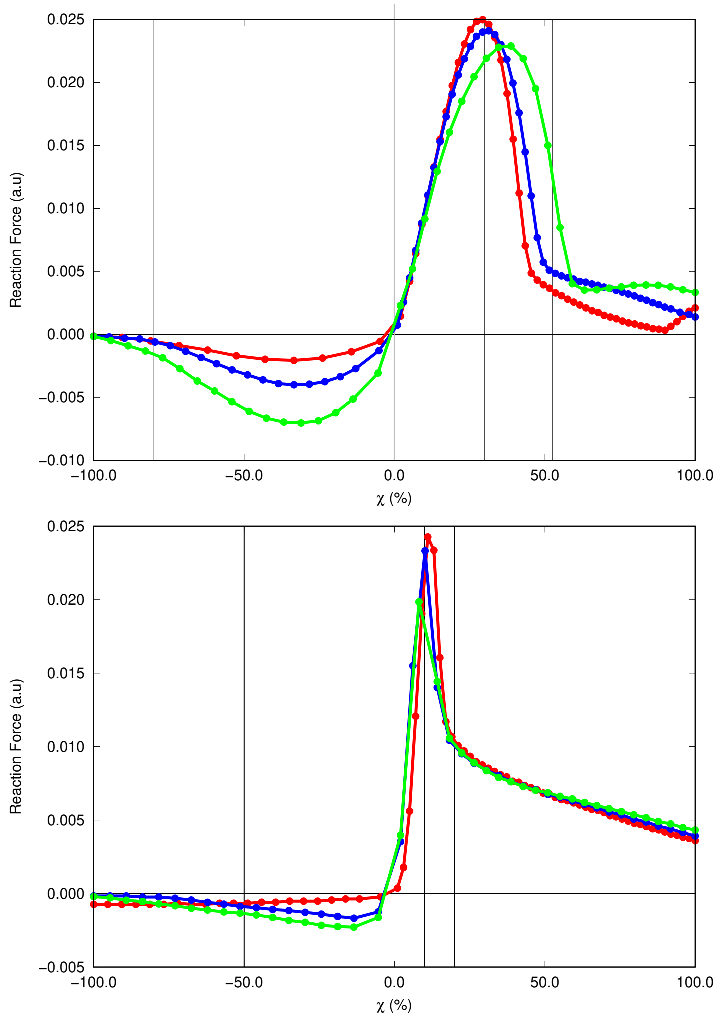

Figure S11: Evolution of the reaction force throughout the substitution (top) and elimination (bottom) reactions under study.

6 Transition State geometries

This section collects the optimized TS geometries involved along the substitution and elimination reactions discussed throughout the manuscript. All geometries are reported in Å.

6.1 Substitution reactions

Atom	X	Y	Z
C	0.88467	-0.13806	-0.00002
Br	-1.35708	-0.12812	0.00000
H	1.01437	-0.69676	-0.91556
H	1.01438	-0.69682	0.91548
C	1.24108	1.31956	0.00001
O	3.16188	-0.58535	-0.00002
H	3.40796	-1.51781	0.00020
H	0.83721	1.81559	-0.89011
H	2.33718	1.35820	0.00004
H	0.83717	1.81556	0.89015

Table S3: Optimized geometry of the TS involved in the gas phase substitution reaction between CH_3CH_2Br and OH^-. Calculations performed at the M06-2X/aug-cc-pVDZ level of theory.

Atom	X	Y	Z
C	0.82818	-0.00873	-0.05584
Br	-1.52350	0.00443	-0.14940
H	0.89253	-0.00545	-1.13108
C	1.04870	-1.29246	0.69364
C	1.08011	1.27437	0.67852
O	3.06815	0.07702	-0.66977
H	3.33497	-0.55310	-1.34979
H	0.75975	2.13503	0.08374
H	2.16495	1.32464	0.83640
H	0.54048	1.27616	1.63359
H	0.76897	-2.15770	0.08359
H	0.45262	-1.30023	1.61445
H	2.12113	-1.32951	0.91825

Table S4: Optimized geometry of the TS involved in the gas phase substitution reaction between $(CH_3)_2CHBr$ and OH^-. Calculations performed at the M06-2X/aug-cc-pVDZ level of theory.

Atom	X	Y	Z
C	0.86725	-0.00009	-0.00048
Br	-1.75306	0.00015	-0.00258
C	0.93496	-1.30654	-0.74106
C	0.99106	-0.01793	1.48813
C	0.93613	1.32407	-0.70927
O	3.28583	-0.00379	0.05683
H	3.80739	0.02780	-0.75466
H	0.65412	1.21494	-1.76168
H	1.97443	1.65976	-0.61886
H	0.25732	2.03773	-0.23338
H	0.52115	-0.91459	1.90351
H	0.52373	0.86999	1.92480
H	2.07348	-0.02148	1.68377
H	0.64263	-1.17344	-1.78785
H	0.26373	-2.03449	-0.27616
H	1.97602	-1.63831	-0.66784

Table S5: Optimized geometry of the TS involved in the gas phase substitution reaction between $(CH_3)_3CBr$ and OH^-. Calculations performed at the M06-2X/aug-cc-pVDZ level of theory.

6.2 Elimination reactions

Atom	X	Y	Z
C	-0.57687	-0.32091	-0.00082
H	-0.70674	-0.92600	-0.89927
C	-1.37081	0.92115	0.00014
Br	1.49234	-0.05248	0.00007
H	-0.70671	-0.92761	0.89660
H	-1.19706	1.52443	-0.90007
H	-1.19696	1.52272	0.90139
H	-2.49320	0.47082	-0.00004
O	-3.70918	-0.42862	0.00108
H	-4.57171	0.00007	-0.00570

Table S6: Optimized geometry of the TS involved in the gas phase elimination reaction between CH_3CH_2Br and OH^-. Calculations performed at the M06-2X/aug-cc-pVDZ level of theory.

Atom	X	Y	Z
C	0.51385	0.05393	-0.24600
H	0.55638	-0.00712	-1.33576
C	1.22372	-1.04628	0.41296
Br	-1.62991	-0.08332	-0.04924
C	0.87767	1.42786	0.26081
H	1.96374	1.52140	0.10366
H	0.66342	1.50543	1.33418
H	0.33746	2.21758	-0.27249
H	0.96837	-2.03111	0.00490
H	1.09448	-1.03051	1.50324
H	2.39213	-0.72424	0.13679
O	3.63130	-0.05570	-0.24107
H	4.32909	-0.70262	-0.38900

Table S7: Optimized geometry of the TS involved in the gas phase elimination reaction between $(CH_3)_2CHBr$ and OH^-. Calculations performed at the M06-2X/aug-cc-pVDZ level of theory.

Atom	X	Y	Z
C	-0.48390	0.00791	0.09093
C	-0.75489	-1.26998	0.85352
C	-1.12373	0.04396	-1.22551
Br	1.72951	-0.00360	-0.11596
C	-0.73362	1.25026	0.91683
H	-1.82414	1.28211	1.06958
H	-0.41893	2.14841	0.37371
H	-0.21053	1.20677	1.87889
H	-0.45395	-2.14454	0.26635
H	-1.84592	-1.29546	1.00278
H	-0.23264	-1.28327	1.81682
H	-0.90426	-0.84229	-1.83209
H	-0.90885	0.96426	-1.78143
H	-2.31719	0.02221	-0.85121
O	-3.56916	-0.04381	-0.12402
H	-4.28613	0.22554	-0.70709

Table S8: Optimized geometry of the TS involved in the gas phase elimination reaction between $(CH_3)_3CBr$ and OH^-. Calculations performed at the M06-2X/aug-cc-pVDZ level of theory.

7 Ionization potentials

The following table collects the DFT estimated ionization potentials (IP) of the different atoms or fragments involved in the studied chemical transformations.

Fragment	Initial	Final	IP (kcal/mol)
C	C (0)	C (+1)	+260.66
H	H (0)	H (+1)	+312.44
Br	Br (-1)	Br (0)	+81.56
OH	OH (-1)	OH (0)	+37.52
CH_3CH_2	CH_3CH_2 (0)	CH_3CH_2 (+1)	+209.98
CH_2CH_2	CH_2CH_2 (0)	CH_2CH_2 (+1)	+293.21
CH_2CH_2	CH_2CH_2 (-1)	CH_2CH_2 (0)	+46.12
$(CH_3)_2CH$	$(CH_3)_2CH$ (0)	$(CH_3)_2CH$ (+1)	+191.30
CH_3CH_2CH	CH_3CH_2CH (0)	CH_3CH_2CH (+1)	+278.57
CH_3CH_2CH	CH_3CH_2CH (-1)	CH_3CH_2CH (0)	+47.72
$(CH_3)_3C$	$(CH_3)_3C$ (0)	$(CH_3)_3C$ (+1)	+181.26
$(CH_3)_2CH_2C$	$(CH_3)_2CH_2C$ (0)	$(CH_3)_2CH_2C$ (+1)	+266.26
$(CH_3)_2CH_2C$	$(CH_3)_2CH_2C$ (-1)	$(CH_3)_2CH_2C$ (0)	+41.03

Table S9: Estimated Ionization Potentials computed in the gas phase at the M06-2X/aug-cc-pVDZ level of theory.

8 Color code used along the Supporting Information

For the sake of simplicity and convenience, the same color code used in the main manuscript will be employed in all the supplementary materials and figures. Thus, the reactions involving different substrates are colored according to the following scheme: red [CH_3CH_2-], blue [$(CH_3)_2CH$-] and green [$(CH_3)_3C$-].

References

[1] A. Martín Pendás and E. Francisco, *Promolden. A QTAIM/IQA code (Avaliable from the authors upon request).*

[2] P. Politzer, A. Toro-Labbé, S. Gutiérrez-Oliva, B. Herrera, P. Jaque, M. C. Concha and J. S. Murray, *J. Chem. Sci*, 2005, **117**, 467–472.
